# Critical roles of microRNA-141-3p and CHD8 in hypoxia/reoxygenation-induced cardiomyocyte apoptosis

**DOI:** 10.1186/s13578-020-00384-5

**Published:** 2020-02-21

**Authors:** Bifeng Yao, Xiaoya Wan, Xinbin Zheng, Ting Zhong, Jia Hu, Yu Zhou, Anna Qin, Yeshuo Ma, Deling Yin

**Affiliations:** 1 Xiangya School of Pharmaceutical Science, Central South University, Changsha, 410008 Hunan China; 2 Department of Neurology, Renmin Hospital of Wuhan University, Wuhan, 430060 Hubei China; 3 Department of Internal Medicine, College of Medicine, East Tennessee State University, Johnson City, TN 37604 USA

**Keywords:** MiR-141-3p, CHD8, Cardiomyocyte, Apoptosis, Hypoxia/reoxygenation, P21

## Abstract

**Background:**

Cardiovascular diseases are currently the leading cause of death in humans. The high mortality of cardiac diseases is associated with myocardial ischemia and reperfusion (I/R). Recent studies have reported that microRNAs (miRNAs) play important roles in cell apoptosis. However, it is not known yet whether miR-141-3p contributes to the regulation of cardiomyocyte apoptosis. It has been well established that in vitro hypoxia/reoxygenation (H/R) model can follow in vivo myocardial I/R injury. This study aimed to investigate the effects of miR-141-3p and CHD8 on cardiomyocyte apoptosis following H/R.

**Results:**

We found that H/R remarkably reduces the expression of miR-141-3p but enhances CHD8 expression both in mRNA and protein in H9c2 cardiomyocytes. We also found either overexpression of miR-141-3p by transfection of miR-141-3p mimics or inhibition of CHD8 by transfection of small interfering RNA (siRNA) significantly decrease cardiomyocyte apoptosis induced by H/R. Moreover, miR-141-3p interacts with CHD8. Furthermore, miR-141-3p and CHD8 reduce the expression of p21.

**Conclusion:**

MiR-141-3p and CHD8 play critical roles in cardiomyocyte apoptosis induced by H/R. These studies suggest that miR-141-3p and CHD8 mediated cardiomyocyte apoptosis may offer a novel therapeutic strategy against myocardial I/R injury-induced cardiovascular diseases.

## Introduction

Cardiovascular diseases are currently the leading cause of death in humans [1–5]. The high mortality of cardiac diseases is associated with myocardial ischemia and reperfusion (I/R) [5]. Myocardial I/R is a pathological state featured by an initial limitation on the blood supply to the heart, followed by blood reperfusion and recovery of oxygen supply [6]. The recovery of blood supply and reoxygenation are always associated with increased tissue damage and inflammatory responses, which are known as reperfusion injury [1, 2]. Moreover, I/R injury may affect the treatment efficiency of cardiac diseases, which could cause severe cell dysfunction, such as apoptosis and necrosis [6]. However, cardiomyocytes are terminally differentiated cells that endogenous regenerative capacity of maintaining cell function is insufficient during severe injury [7]. Therefore, inhibition of cardiomyocyte apoptosis may have promised as a therapeutic strategy for I/R injury. Recent studies have made some progress on mechanisms of myocardial I/R injury. However, clinical treatments are accompanied by low cure rate. Therefore, the molecular mechanisms of myocardial I/R injury remain to be further explored.

MicroRNAs (miRNAs) are small, highly conserved, and non-coding RNA, which containing approximately 22 nucleotides [8–10]. MiRNAs promote the degradation of mRNA or inhibit the translation of mRNA by targeting the 3′ untranslated region of mRNA, thereby regulating gene expression at post-transcriptional level [10–12]. MiRNAs are dominant players in different aspects of cardiovascular development [10], including cell apoptosis, cell proliferation, and differentiation [13–15]. Our previous studies by RNA sequencing found that a long non-coding RNA is involved in ischemia and reperfusion injury. Furthermore, we found by database prediction that miR-141-3p is one of the downstream of this long non-coding RNA (data not shown). Previous studies reported that miR-141-3p is associated with glioma cell growth [16], mesenchymal stem cell aging [17], and I/R injury in endothelial cells [18]. However, the roles and mechanisms of miR-141-3p in myocardial I/R injury or in hypoxia/reoxygenation (H/R) in vitro model remain to be elucidated.

Chromodomain helicase DNA-binding protein 8 (CHD8) is a member of ATP-dependent chromatin remodeling protein in the CHD family [19]. CHD8 is related to the development of autism [20, 21], and involved in embryonic development and apoptosis of vascular smooth muscle cells [19, 22]. However, the function of CHD8 in myocardial I/R injury and H/R is not known yet.

P21, the well-known cyclin-dependent kinase inhibitor, is a 165 amino acids protein that confirmed to play an important role in inhibition of cell cycle, suppression of tumor progress and cell apoptosis [23, 24]. Recent studies have reported p21 served as a pro-apoptosis regulator in cardiomyocytes [25]. But the further mechanism still needs to be elucidated.

It has been well established that in vitro H/R model can follow in vivo myocardial I/R injury [26, 27]. In the current study, we performed the hypoxia/reoxygenation model, one that has been widely used to investigate the mechanisms of myocardial I/R injury [26, 27]. We found that the expression of miR-141-3p attenuates cardiomyocyte apoptosis induced by H/R. Additionally, downregulation of CHD8 inhibits cardiomyocyte apoptosis induced by H/R. Furthermore, we found miR-141-3p and CHD8 reduce p21 expression. Thus, our studies may provide a novel potential therapeutic target against myocardial I/R injury-induced cardiovascular diseases.

## Materials and methods

### Cell culture

H9c2 cardiomyocytes were obtained from the Cell Bank of Shanghai Institute of Cell Biology, Chinese Academy of Sciences. The cells were cultured with basic Dulbecco's modified Eagle's medium (DMEM) containing 10% fetal bovine serum. Cultures were incubated at 37 °C and 5% CO_2_ in a fully humidified incubator.

### The hypoxia/reoxygenation cell culture model

To establish the hypoxia/reoxygenation in vitro model, H9c2 cardiomyocytes were cultured with low glucose (1 mg/mL glucose) DMEM and incubated in an oxygen-free atmosphere (95% N_2_ and 5% CO_2_, 37 °C). After 8 h of culture, the cells were changed to a normal culture medium (4.5 mg/mL glucose) and normal atmosphere (95% air and 5% CO_2_, 37 °C) for another 48 h. Cells in the control group were cultured under normal conditions.

### RNA isolation, reverse transcription and real-time quantitative RT-PCR (qRT-PCR) analysis

The qRT-PCR was performed as described previously by our work [28]. Briefly, total RNA in H9c2 cells were extracted using TRIzol reagent (Cwbio, Beijing, China). Reverse transcription 1 μg RNA per sample using miDETECT A Track™ miRNA qRT-PCR Starter Kit (RiboBio Co., Ltd, Guangzhou, China) to synthetic cDNA to detect miR-141-3p. While using PrimeScript™ RT reagent Kit (Perfect Real Time) (Takara, Japan) for other mRNAs. PCR analysis was executed using iTaq™ universal SYBR® Green Supermix (Bio-Rad, Hercules, CA). Expression level of miR-141-3p was detected using U6 as internal control, while expression levels of other mRNAs were detected using GAPDH as internal control. The primer sequences used are listed in Table 1.

**Table 1:** Primers used for qRT-PCR

Gene	Forward primer sequence (5′–3′)	Reverse primer sequence (5′–3′)	NCBI accession ID	Length of the amplicons
miR-141-3p	The primer sequences are proprietary information of the company. (RiboBio Co., Ltd, Guangzhou, China)			
U6	ATTGGAACGATA CAGAGAAGATT	GGAACGCTT CACGAATTTG	K00784	70 bp
CHD8	CCTCACGCAC TGCTTCACCATC	CTCCTAGCCACC ACCTCATCCTC	NM_001347661	133 bp
GAPDH	GGTGGACCTCA TGGCCTACA	CTCTCTTGCTCTC AGTATCCTTGCT	NM_017008.4	84 bp

### Western blot analysis

Western blot analysis was performed as previous described [29]. Briefly, total protein was extracted from the cells using RIPA lysis buffer (Cwbio, Beijing, China), which was added with Protease Inhibitor Cocktail (1%, v/v) (Cwbio, Beijing, China). Then proteins were separated by 8% and 12% SDS-PAGE gels and electrotransfer onto Immobilon PVDF membranes (Merck KGaA, Darmstadt, Germany). After blocking the membranes with fat free milk, the membranes were incubated overnight at 4 °C with primary antibodies (Table 2). After incubation with secondary antibodies (goat anti-rabbit IgG, Proteintech, China), membranes were then imaged by an enhanced chemiluminescent detection kit (Cwbio, Beijing, China).

**Table 2:** Antibodies used for Western blotting

Name	Description	Manufacturer
Anti-β-actin	Rabbit monoclonal, 43 kDa	Proteintech (20,536–1-AP)
Anti-Caspase-3	Rabbit monoclonal, 34 kDa	CST (#9662S)
Anti-Cleaved caspase-3	Rabbit monoclonal, 16 kDa	CST (#9664S)
Anti-CHD8	Rabbit monoclonal, 290 kDa	CST (#77,694)
Anti-p21	Rabbit monoclonal, 21 kDa	Abcam (109,199)

### Cell apoptosis detection by flow cytometric analysis

Flow cytometric analysis was performed as previous described by us [30]. Apoptotic cells were determined by flow cytometer (Becton, Dickinson and Company, CA, USA) and the percentage of apoptotic cells was determined by FITC Annexin V Apoptosis Detection Kit I (BD Biosciences, Franklin Lakes, NJ, USA). Cells were gathered according to the manufacturer’s instructions and cells were rinsed twice with cold PBS and then resuspended in binding buffer at a concentration of 1 × 10^6^ cells/ mL. 100 µl of the solution (1 × 10^5^ cells) was transferred to per culture tube, 5 µl of FITC Annexin V and 5 µl PI were added subsequently. Then, the cells were gently vortexed and incubated for 15 min at RT in the dark. Finally, 400 µl of binding buffer were putted into each tube and analyzed by flow cytometry within 1 h.

### Transfection of overexpression mimics or siRNA

Mimics and small interfering RNA transfection (siRNA) were performed as described previously by us [31]. Briefly, H9c2 cells were cultured in 6-wells plates. To overexpression of miR-141-3p, when H9c2 cells were visualized at 40% density, transient transfected with miR-141-3p mimics or miR-141-3p negative control (miR-141-3p NC) (GenePharma, Shanghai, China) using Lipofectamine™ 2000 (Invitrogen, Carlsbad, CA, USA) according to the manufacturer’s instruction. After 48 h culture, H9c2 cells were harvested for H/R treatment or further study. The miR-141-3p mimics and miR-141-3p NC sequences used are as follows:

miR-141-3p mimics: 5′-UAACACUGUCUGGUAAAGAUGG-3′;

miR-141-3p negative control: 5′-ACGUGACACGUUCGGAGAATT-3′;

Similarly, to knockdown the expression of CHD8, siRNA oligonucleotides against CHD8 and negative control siRNA (NC-Si) were designed and synthesized by the RiboBio Co., Ltd. (Guangzhou, China). H9c2 cells were transient transfected with a mixture of siRNA using Lipofectamine™ 2000 (Invitrogen, Carlsbad, CA, USA) after observed at 40% density. After 48 h culture, the knockdown efficiency was determined by Western blot analysis and for further study. The siRNA sequences used are as follows:

CHD8-siRNA: 5′-CGATGTTACTGGTCCAATA-3′;

Negative control siRNA: 5′-TTCTCCGAACGTGTCACGT-3′.

### RNA immunoprecipitation (RIP)

RIP was carried out using CHD8 antibody and the IgG antibody, which was served as negative control [32]. Briefly, the cells were harvested by RIPA lysis buffer containing Protease Inhibitor Cocktail and RiboLock RNase inhibitor (1%, v/v) (Thermo Fisher Scientific, Waltham, MA, USA) according to the manufacture’s instruction. Obtained samples were incubated for 1 h at 4 °C. Then, Protein A/G PLUS-Agarose was added into the samples and rotated overnight at 4 °C. Beads were washed for 4 times using lysis buffer. Finally, divided the samples into two equal parts respectively. One for Western blot analysis to determined expression level of CHD8, and the other for qRT-PCR analysis to detect expression level of miR-141-3p.

### Statistical analysis

Graphpad Prism 5.01 was used to analyze data in this study. The results were presented as mean ± SD. The data were analyzed using one-way analysis of variance and Student’s t-test. A value of *P* < 0.05 was considered to be statistically significant.

## Results

### MiR-141-3p is downregulated by hypoxia/reoxygenation (H/R) in H9c2 cardiomyocytes

**Fig. 1:**
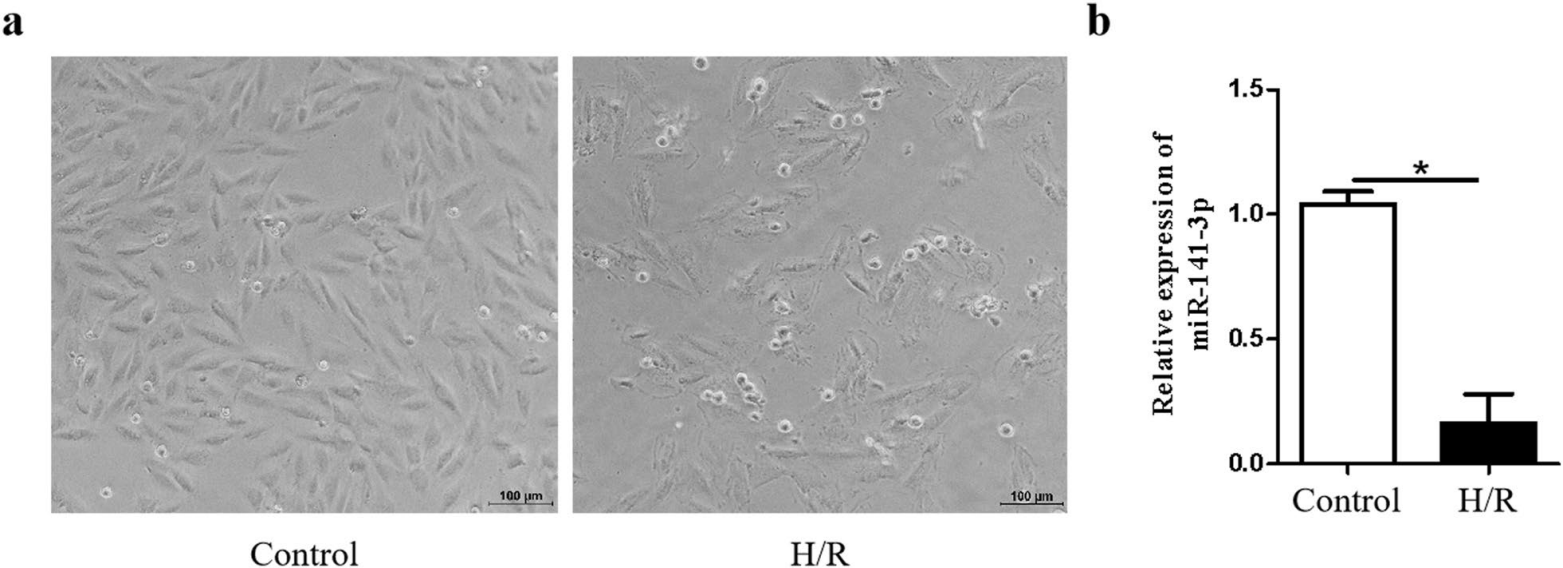
MiR-141-3p is downregulated by hypoxia/reoxygenation (H/R) in H9c2 cardiomyocytes. H9c2 cardiomyocytes were subjected to hypoxia treatment for 8 h with low glucose (1 mg/mL glucose) DMEM and oxygen-free atmosphere (95% N_2_ and 5% CO_2_, 37 °C), then reoxygenated for 48 h with normal culture medium (4.5 mg/mL glucose) and normal atmosphere (95% air and 5% CO_2_, 37 °C). **a** The alteration of morphology was observed by microscope. **b** The expression of miR-141-3p was determined by qRT-PCR. N = 3 per group. *P < 0.05, compared with control group

To investigate whether miR-141-3p plays a role in H/R-induced injury in cardiomyocytes. We first evaluated the morphological alterations of H9c2 cardiomyocytes following H/R and observed that H/R promoted cell damage and reduced the number of H9c2 cardiomyocytes (Fig. 1a). We then determined the expression level of miR-141-3p following H/R treatment by real-time quantitative RT-PCR (qRT-PCR). As shown in Fig. 1b, the expression level of miR-141-3p was dramatically decreased in H9c2 cardiomyocytes following H/R treatment, indicating that miR-141-3p might be involved in cardiomyocyte H/R injury.

### Overexpression of miR-141-3p dramatically decreases H/R-induced H9c2 cardiomyocyte apoptosis

**Fig. 2:**
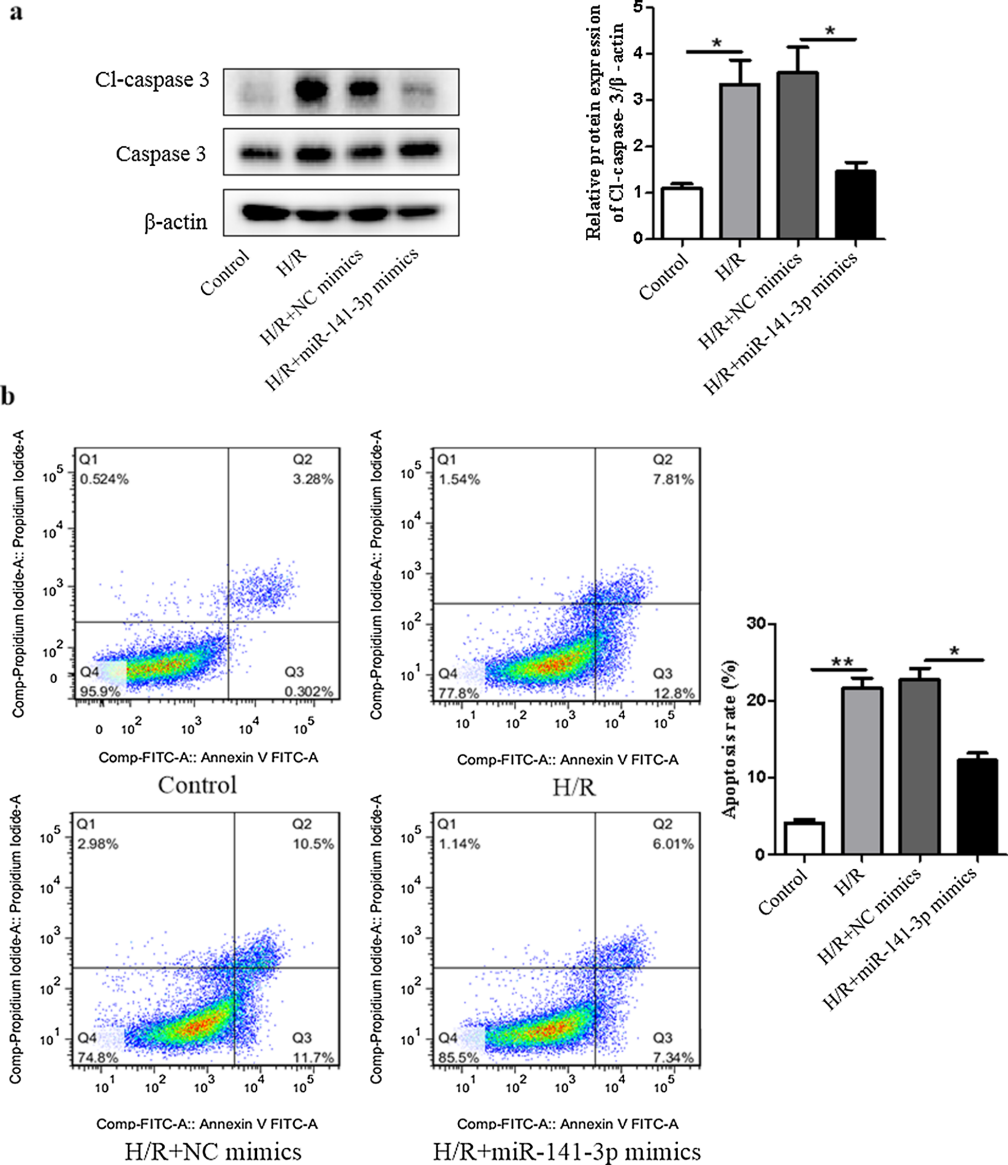
Overexpression of miR-141-3p decreases H/R induced apoptosis in H9c2 cardiomyocytes. H9c2 cardiomyocytes were transfected with miR-141-3p mimics or miR-141-3p NC for 6 h in low glucose DMEM, then cultured for another 48 h in basic DMEM. Transfected H9c2 cardiomyocytes were subjected to hypoxia treatment for 8 h, then reoxygenated for 48 h as in Fig. 1. **a** The cleaved caspase-3 (Cl-caspase-3) and caspase-3 were measured by Western blot analysis. **b** Apoptotic cells were determined by flow cytometric analysis. N = 3 per group. *NC* negative control. *P < 0.05, **P < 0.01, compared with indicated groups

To determine whether miR-141-3p contributed to H/R induced cardiomyocyte apoptosis, we examined the alterations of apoptotic marker cleaved caspase-3 (Cl-caspase-3) protein and also performed flow cytometric analysis in H9c2 cardiomyocytes after transfection with miR-141-3p mimics. We found that overexpression of miR-141-3p significantly inhibited H/R-induced Cl-caspase-3 (Fig. 2a) and apoptosis in H9c2 cardiomyocytes (Fig. 2b). Taken together, these results reveal that miR-141-3p plays a critical role in cardiomyocyte apoptosis induced by H/R.

### H/R induces the expression of CHD8 in H9c2 cardiomyocytes

**Fig. 3:**
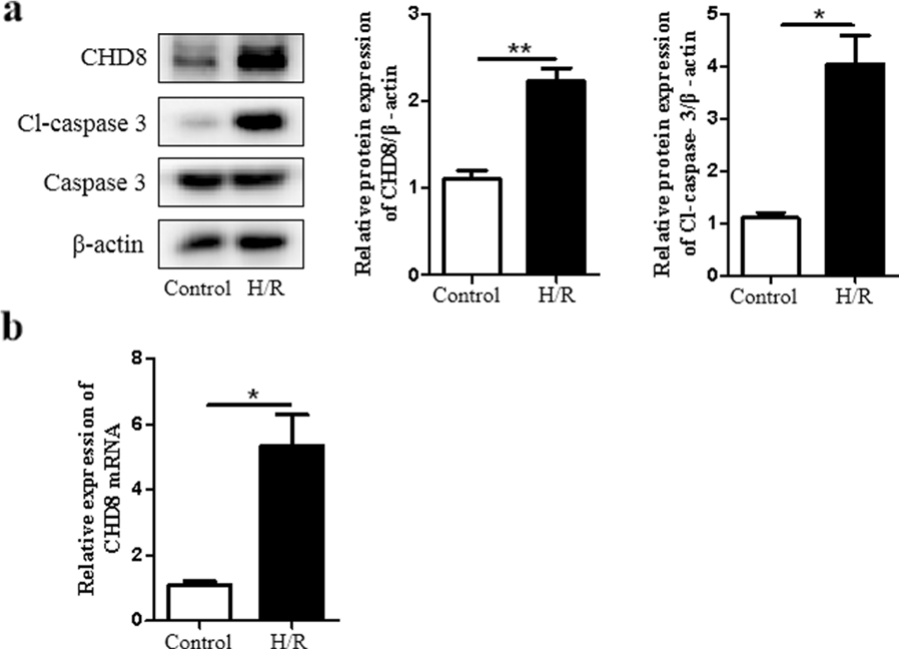
CHD8 is upregulated by H/R in H9c2 cardiomyocytes. H9c2 cardiomyocytes were subjected to hypoxia treatment for 8 h, then reoxygenated for 48 h as in Fig. 1. **a** The expression of CHD8, Cl-caspase-3, and caspase-3 was examined by Western blot. **b** CHD8 miRNA expression was determined by qRT-PCR. N = 3 per group. *P < 0.05, **P < 0.01, compared with control group

We then evaluate whether the expression of CHD8 was altered following H/R. Our results showed that H/R significantly increased CHD8 expression in both protein and mRNA levels in H9c2 cardiomyocytes (Fig. 3a, b), indicating that CHD8 may participate in cardiomyocyte apoptosis induced by H/R.

### Downregulation of CHD8 inhibits H9c2 cardiomyocyte apoptosis induced by H/R

**Fig. 4:**
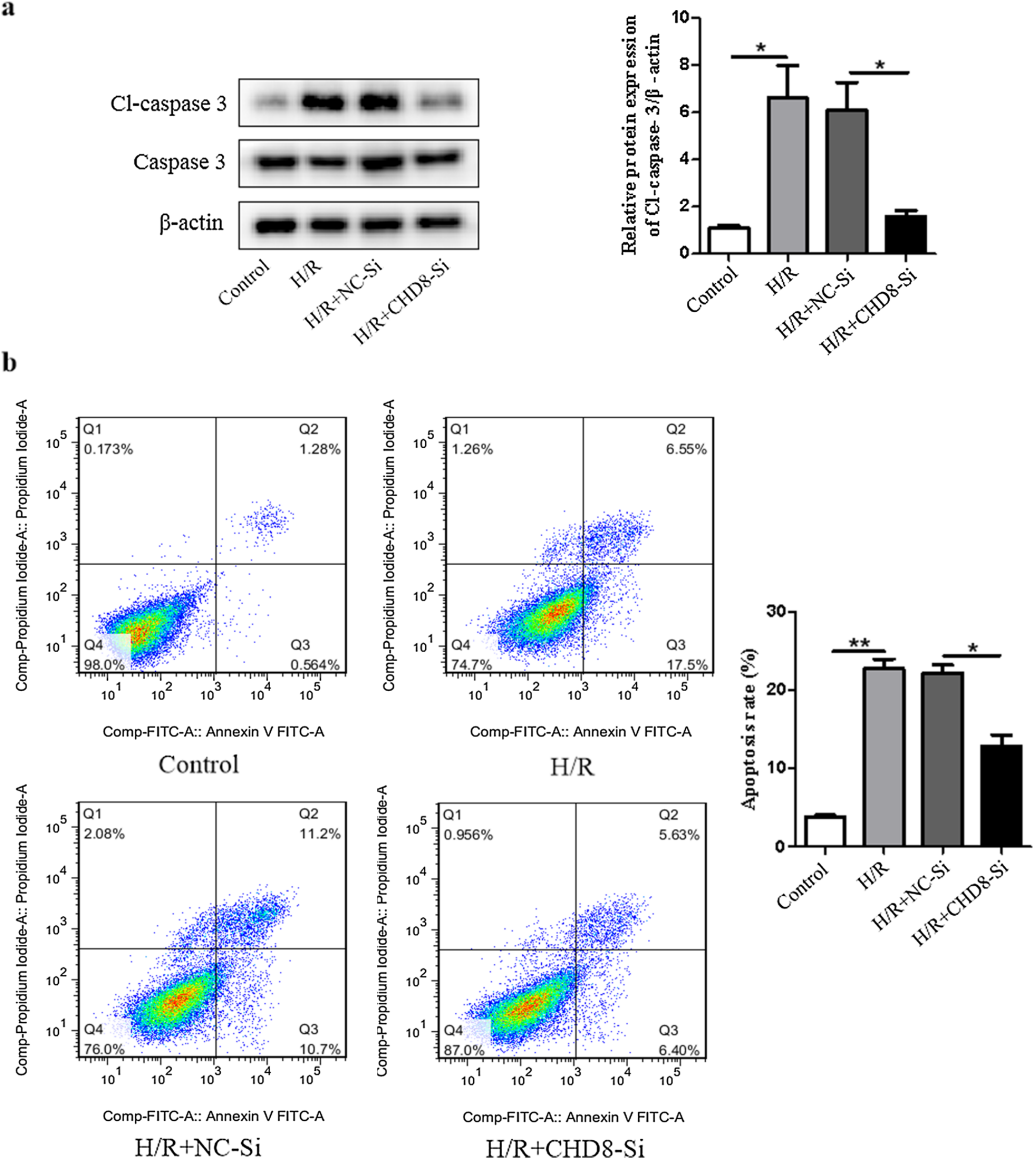
Knockdown of CHD8 inhibits apoptosis induced by H/R in H9c2 cardiomyocytes. H9c2 cardiomyocytes were transfected with CHD8-siRNA (CHD8-Si) or negative control-siRNA (NC-Si) for 6 h in low glucose DMEM, then cultured for another 48 h in basic DMEM. Transfected H9c2 cardiomyocytes were subjected to hypoxia treatment for 8 h, then reoxygenated for 48 h as in Fig. 1. **a** The expression of Cl-caspase-3 and caspase-3 was determined by Western blot. **b** Apoptotic cells were determined by flow cytometric analysis. N = 3 per group. *P < 0.05, **P < 0.01, compared with indicated groups

To assess the role of CHD8 in H/R induced apoptosis, H9c2 cardiomyocyte were transfected with small interfering RNA (siRNA). As shown in Fig. 4a, inhibition of CHD8 significantly decreased the expression level of Cl-caspase-3 induced by H/R. Moreover, we performed flow cytometry analysis and found that inhibition of CHD8 dramatically diminished the number of cardiomyocyte apoptosis induced by H/R (Fig. 4b). These results demonstrated that knockdown of CHD8 could ameliorate H/R induced cardiomyocyte apoptosis.

### MiR-141-3p interacts with CHD8 in H9c2 cardiomyocytes

**Fig. 5:**
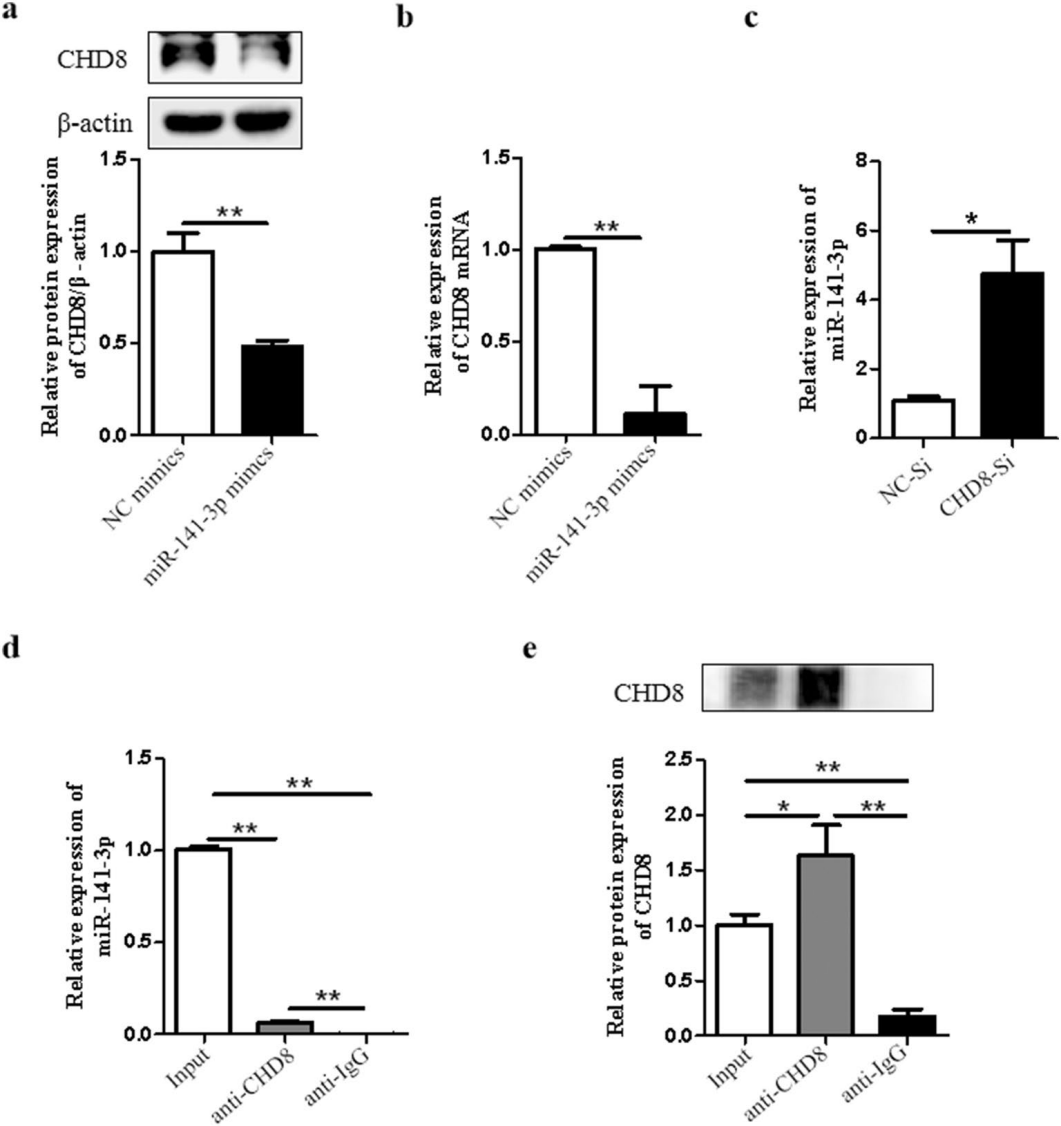
MiR-141-3p interacts with CHD8 in H9c2 cardiomyocytes. H9c2 cardiomyocytes were transfected with miR-141-3p mimics or miR-141-3p NC for 6 h in low glucose DMEM, then cultured for another 48 h in basic DMEM. CHD8 expression was examined by Western blot (**a**) and qRT-PCR (**b**). **c** H9c2 cardiomyocytes were transfected with CHD8-siRNA (CHD8-Si) or negative control-siRNA (NC-Si) for 6 h in low glucose DMEM, then cultured for another 48 h in basic DMEM. The expression of miR-141-3p was examined by qRT-PCR. The relationship between miR-141-3p and CHD8 was determined by RIP analysis (d, e). Collected H9c2 cardiomyocytes were extracted by RIPA lysis buffer. Obtained samples were pre-incubated with protein A/G PLUS-agarose. The samples were then divided into two parts equally and incubated with IgG and CHD8 overnight separately. Washed samples and then divided each sample into two parts, one for Western blot analysis and the other for qPCR analysis. **d** The expression level of miR-141-3p was measured by qRT-PCR. **e** The protein expression level of CHD8 was examined by Western blot analysis. N = 3 per group. *NC* negative control. *P < 0.05, **P < 0.01, compared with indicated groups

We then examined whether there is interaction between miR-141-3p and CHD8. We showed that overexpression of miR-141-3p reduced the expression of CHD8 in both protein and mRNA levels (Fig. 5a, b). Interestingly, inhibition of CHD8 increased the expression level of miR-141-3p (Fig. 5c). Therefore, we examined the relationship between them by RIP analysis. Collected H9c2 cardiomyocytes were extracted by RIPA lysis buffer. As shown in Fig. 5d, e, qRT-PCR and Western blot analysis showed the relationship between miR-141-3p and CHD8. In addition, we didn’t find complementary base pairs of miR-141-3p and CHD8 mRNA by searching for TargetScan (data not shown). Collectively, these findings suggested that there is an indirect interaction between miR-141-3p and CHD8 in H9c2 cardiomyocytes.

### MiR-141-3p and CHD8 can reduce the expression of p21 in H9c2 cardiomyocytes

**Fig. 6:**
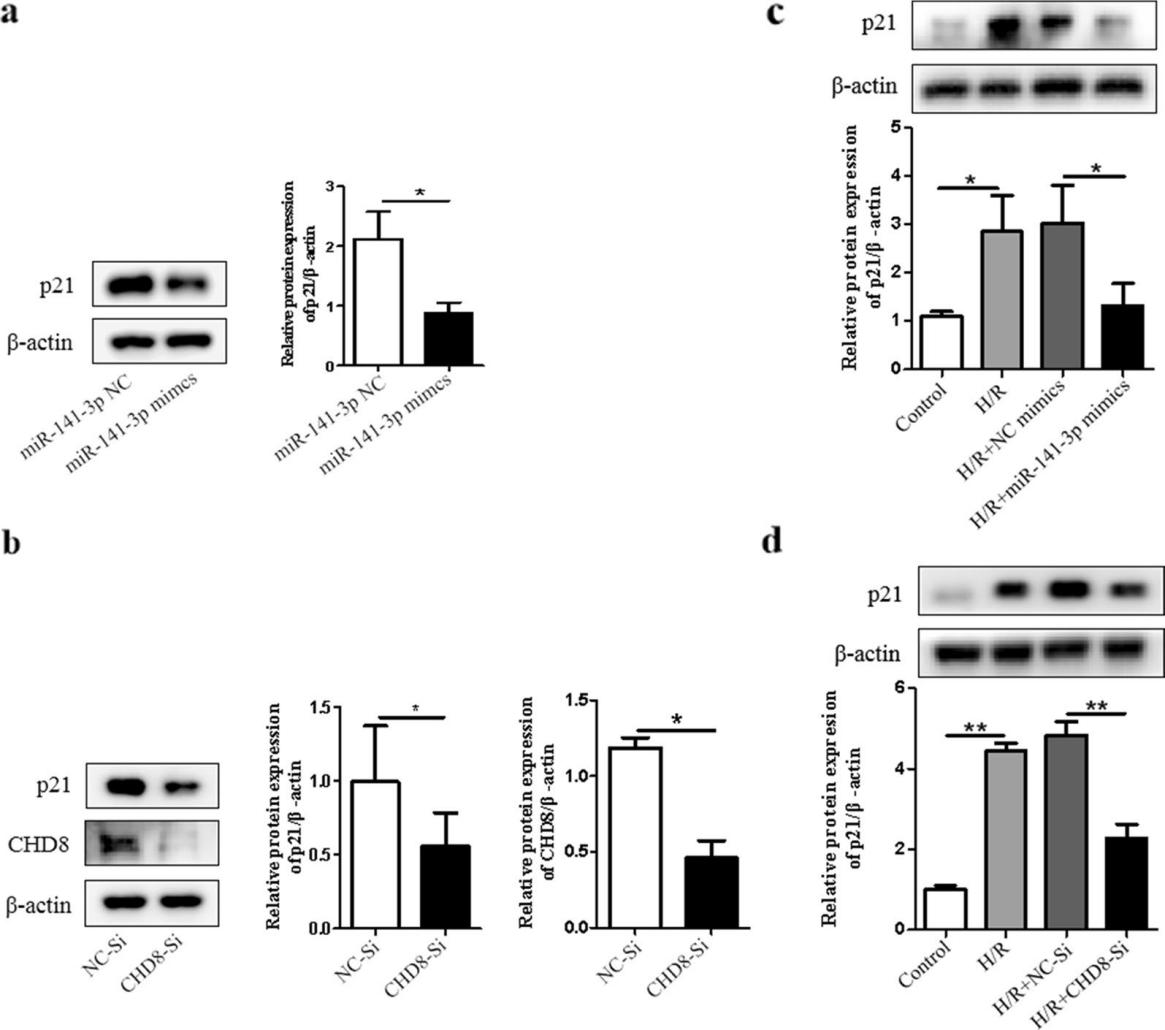
MiR-141-3p and CHD8 reduce the expression level of p21 in H9c2 cardiomyocytes. H9c2 cardiomyocytes were transfected with miR-141-3p mimics or CHD8-Si for 6 h in low glucose DMEM, then cultured for another 48 h in basic DMEM. **a** P21 expression was examined by Western blot. **b** The expression of p21 and CHD8 was determined by Western blot. After transfected with miR-141-3p mimics or CHD8-Si as a, b, H9c2 cardiomyocytes were subjected to hypoxia treatment for 8 h, then reoxygenated for 48 h as in Fig. 1. **c**, **d** P21 expression was examined by Western blot. N = 3 per group. *P < 0.05, **P < 0.01, compared with indicated groups

Previous study has shown that p21 could act as an apoptosis promoting regulator [25]. We therefore investigated that whether miR-141-3p or CHD8 plays a role in alteration of p21 expression. Notably, we found that either overexpression of miR-141-3p or inhibition of CHD8 significantly decreased the expression of p21 (Fig. 6a, b). We further determined whether miR-141-3p or CHD8 had effect on p21 expression following H/R. Our results showed that the expression of p21 was decreased after transfection with miR-141-3p mimics or CHD8-Si following H/R treatment (Fig. 6c, d). Therefore, these results suggest that p21 contributes to miR-141-3p and CHD8 mediated signaling in H/R.

## Discussion

To the best of our knowledge, the results of this study reveal for the first time that miR-141-3p is downregulated and exerts as a protective regulator against H/R induced cardiomyocyte apoptosis. Subsequently, CHD8 is verified to act as a pro-apoptotic molecular in H/R induced cardiomyocyte apoptosis. Meanwhile, miR-141-3p and CHD8 regulate the expression of p21. These studies reveal that miR-141-3p, a potential target of myocardial I/R injury, may provide a novel therapeutic strategy on cardiac diseases, which based on interacting with CHD8.

Myocardial I/R injury has become a prominent problem that influences therapeutical effect of reperfusion therapy on ischemic myocardium [33]. Further, reperfusion accelerates the process of apoptosis induced by ischemia itself [34]. Due to the apoptosis of cardiomyocytes in the ischemic site occurs immediately, it causes enrichment of reactive oxygen species with reperfusion progressing, which eventually aggravates the degree of apoptosis [35, 36]. Thus, it’s well established that ameliorating apoptosis plays a pivotal role against I/R injury.

Increasing number of miRNAs, such as miR-25 and miR-762 modulate the expression of key molecular associated with apoptosis in myocardial I/R injury [37, 38]. Previous studies have shown that miR-141-3p alters the expression of p53 as a reason of promoting glioblastoma progression and temozolomide resistance [16]. MiR-141-3p also has impact on mesenchymal stem cell senescence by directly targeting ZMPSTE24 [17]. MiR-141 decreases myocardial I/R injury in endothelium by regulating expression of ICAM-1 [18]. In our study, the results showed that the expression of miR-141-3p is significantly downregulated and overexpression of miR-141-3p alleviates the cardiomyocyte apoptosis induced by H/R.

CHD8 is a protective molecular in apoptosis. It decreased p53-mediated apoptosis during early embryogenesis [22]. In addition, it was also confirmed to act as an inhibitor of apoptosis in A10 vascular smooth muscle cells [19]. Surprisingly, the results of our study showed that the expression of CHD8 is increased and it promotes H9c2 cardiomyocyte apoptosis following H/R. Our study unveiled for the first time that CHD8 plays an important role in cardiomyocyte apoptosis, which also indicates its significant function in cardiac diseases. Our results from Western blot, qPCR, and RIP analysis all showed that miR-141-3p and CHD8 have effects on each other. However, we could not find complementary sequences between them by bioinformatic analysis. Our data suggest that other protein(s) may mediate between them, which will be investigated in our future studies.

Previous studies have reported that p21 served as a regulator of anti-apoptosis [39, 40], while other studies verified its pro-apoptotic effect [25]. It inhibits the activation of cell cycle which is necessary to initiate apoptosis. Otherwise, under certain conditions, p21 promotes cell apoptosis in either p53-dependent or p53-independent mechanisms [41]. In our current study, the results reveal for the first time that the expression of p21 is regulated by miR-141-3p and CHD8. But it still remains further studies that whether the effects of miR-141-3p and CHD8 in H/R induced apoptosis is through p21. We recognize that the limitation of this study was that only a single cell line and more cell lines will be determined in our future studies.

## Conclusion

In summary, this is the first study to provide evidence illustrating that the expression of miR-141-3p attenuates cardiomyocyte apoptosis induced by H/R by interacting with CHD8. Our findings indicated that the mechanism underlying miR-141-3p and CHD8 interaction in H/R induced cardiomyocyte apoptosis may provide a novel therapeutic strategy against myocardial I/R injury-induced cardiovascular diseases.

## Data Availability

All relevant data are swithin this published paper.

## Abbreviations

I/R: Ischemia/reperfusion
miRNAs: MicroRNAs
H/R: Hypoxia/reoxygenation
CHD8: Chromodomain helicase DNA-binding protein 8
DMEM: Basic Dulbecco's modified Eagle's medium
qRT-PCR: Quantitative real-time polymerase chain reaction
siRNA: Small interfering RNA
NC-Si: Negative control siRNA
RIP: RNA immunoprecipitation
Cl-caspase-3: Cleaved caspase-3

## References

[1] Yellon DM, Hausenloy DJ (2007). Myocardial reperfusion injury. N Engl J Med.

[2] Yellon DM, Hausenloy DJ (2013). Myocardial ischemia-reperfusion injury: a neglected therapeutic target. J Clin Invest.

[3] Aurora AB, Mahmoud AI, Luo X, Johnson BA, van Rooij E, Matsuzaki S (2012). MicroRNA-214 protects the mouse heart from ischemic injury by controlling Ca^2+^ overload and cell death. J Clin Invest.

[4] Melo Z, Ishida C, Goldaraz MP, Rojo R, Echavarria R (2018). Novel roles of non-coding RNAs in opioid signaling and cardioprotection. Noncoding RNA.

[5] González-Montero J, Brito R, Gajardo AI, Rodrigo R (2018). Myocardial reperfusion injury and oxidative stress: therapeutic opportunities. World J Cardiol.

[6] Eltzschig HK, Eckle T (2011). Ischemia and reperfusion–from mechanism to translation. Nat Med.

[7] Chavakis E, Koyanagi M, Dimmeler S (2010). Enhancing the outcome of cell therapy for cardiac repair progress from bench to bedside and back. Circulation.

[8] Van der Kwast RVCT, Quax PHA, Nossent AY (2019). An emerging role for isomiRs and the microRNA epitranscriptome in neovascularization. Cells.

[9] Matsuyama H, Suzuki HI (2019). Systems and synthetic microRNA biology: from biogenesis to disease pathogenesis. Int J Mol Sci.

[10] Cordes KR, Srivastava D (2009). MicroRNA regulation of cardiovascular development. Circ Res.

[11] Mens MMJ, Ghanbari M (2018). Cell cycle regulation of stem cells by microRNAs. Stem Cell Rev Rep.

[12] Gottlieb RA, Pourpirali S (2016). Lost in translation: miRNAs and mRNAs in ischemic preconditioning and ischemia/reperfusion injury. J Mol Cell Cardiol.

[13] Menbari MN, Rahimi K, Ahmadi A, Elyasi A, Darvishi N, Hosseini V (2019). MiR-216b-5p inhibits cell proliferation in human breast cancer by down-regulating HDAC8 expression. Life Sci.

[14] Lee SY, Yang J, Park JH, Shin HK, Kim WJ, Kim SY (2020). The MicroRNA-92a/Sp1/MyoD axis regulates hypoxic stimulation of myogenic lineage differentiation in mouse embryonic stem cells. Mol Ther.

[15] Feng X, Xiong W, Yuan M, Zhan J, Zhu X, Wei Z (2019). Down-regulated microRNA-183 mediates the Jak/Stat signaling pathway to attenuate hippocampal neuron injury in epilepsy rats by targeting Foxp1. Cell Cycle.

[16] Zhou X, Wu W, Zeng A, Nie E, Jin X, Yu T (2017). MicroRNA-141-3p promotes glioma cell growth and temozolomide resistance by directly targeting p53. Oncotarget.

[17] Yu KR, Lee S, Jung JW, Hong IS, Kim HS, Seo Y (2013). MicroRNA-141-3p plays a role in human mesenchymal stem cell aging by directly targeting ZMPSTE24. J Cell Sci.

[18] Liu RR, Li J, Gong JY, Kuang F, Liu JY, Zhang YS (2015). MicroRNA-141 regulates the expression level of ICAM-1 on endothelium to decrease myocardial ischemia-reperfusion injury. Am J Physiol Heart Circ Physiol.

[19] Rodenberg JM, Hoggatt AM, Chen M, Touw K, Jones R, Herring BP (2010). Regulation of serum response factor activity and smooth muscle cell apoptosis by chromodomain helicase DNA-binding protein 8. Am J Physiol Cell Physiol.

[20] Kasah S, Oddy C, Basson MA (2018). Autism-linked CHD gene expression patterns during development predict multi-organ disease phenotypes. J Anat.

[21] Xu Q, Liu YY, Wang X, Tan GH, Li HP, Hulbert SW (2018). Autism-associated CHD8 deficiency impairs axon development and migration of cortical neurons. Mol Autism.

[22] Nishiyama M, Oshikawa K, Tsukada Y, Nakagawa T, Iemura S, Natsume T (2009). CHD8 suppresses p53-mediated apoptosis through histone H1 recruitment during early embryogenesis. Nat Cell Biol.

[23] Karimian A, Ahmadi Y, Yousefi B (2016). Multiple functions of p21 in cell cycle, apoptosis and transcriptional regulation after DNA damage. DNA Repair (Amst).

[24] Shamloo B, Usluer S (2019). P21 in cancer research. Cancers (Basel).

[25] Liu X, Zhang C, Qian L, Zhang C, Wu K, Yang C (2015). NF45 inhibits cardiomyocyte apoptosis following myocardial ischemia-reperfusion injury. Pathol Res Pract.

[26] Gao Y, Yin H, Zhang Y, Dong Y, Yang F, Wu X (2019). Dexmedetomidine protects hippocampal neurons against hypoxia/reoxygenation-induced apoptosis through activation HIF-1α/p53 signaling. Life Sci.

[27] Hou Z, Qin X, Hu Y, Zhang X, Li G, Wu J (2019). Longterm exercise-derived exosomal miR-342-5p. Circ Res.

[28] Qin A, Zhong T, Zou H, Wan X, Yao B, Zheng X (2019). Critical role of Tim-3 mediated autophagy in chronic stress induced immunosuppression. Cell Biosci.

[29] Zhou Y, Song Y, Shaikh Z, Li H, Zhang H, Caudle Y (2017). MicroRNA-155 attenuates late sepsis-induced cardiac dysfunction through JNK and β-arrestin 2. Oncotarget.

[30] Zheng X, Zhong T, Ma Y, Wan X, Qin A, Yao B (2019). Bnip3 mediates doxorubicin-induced cardiomyocyte pyroptosis via caspase-3/GSDME. Life Sci.

[31] Liu H, Liu P, Shi X, Yin D, Zhao J (2018). NR4A2 protects cardiomyocytes against myocardial infarction injury by promoting autophagy. Cell Death Discov.

[32] Wang K, Gan TY, Li N, Liu CY, Zhou LY, Gao JN (2017). Circular RNA mediates cardiomyocyte death via miRNA-dependent upregulation of MTP18 expression. Cell Death Differ.

[33] Liu S, He Y, Shi J, Liu L, Ma H, He L (2019). Downregulation of miRNA-30a enhanced autophagy in osthole-alleviated myocardium ischemia/reperfusion injury. J Cell Physiol.

[34] Eefting F, Rensing B, Wigman J, Pannekoek WJ, Liu WM, Cramer MJ (2004). Role of apoptosis in reperfusion injury. Cardiovasc Res.

[35] Hori M, Nishida K (2009). Oxidative stress and left ventricular remodelling after myocardial infarction. Cardiovasc Res.

[36] Sun Y (2009). Myocardial repair/remodelling following infarction: roles of local factors. Cardiovasc Res.

[37] Yan K, An T, Zhai M, Huang Y, Wang Q, Wang Y (2019). Mitochondrial miR-762 regulates apoptosis and myocardial infarction by impairing ND2. Cell Death Dis.

[38] Qin X, Gao S, Yang Y, Wu L, Wang L (2019). MicroRNA-25 promotes cardiomyocytes proliferation and migration via targeting Bim. J Cell Physiol.

[39] Gong L, Wen T, Li Z, Wang Y, Wang J, Che X (2019). TNPO2 operates downstream of DYNC1I1 and promotes gastric cancer cell proliferation and inhibits apoptosis. Cancer Med.

[40] Ramachandran R, Saraswathi M (2017). Postconditioning with metformin attenuates apoptotic events in cardiomyoblasts associated with ischemic reperfusion injury. Cardiovasc Ther.

[41] Abbas T, Dutta A (2009). P21 in cancer: intricate networks and multiple activities. Nat Rev Cancer.

